# Stomach as the target organ of *Rickettsia heilongjiangensis* infection in C57BL/6 mice identified by click chemistry

**DOI:** 10.1038/s42003-024-06468-z

**Published:** 2024-06-29

**Authors:** Juan Wang, Li-Feng Du, Ming-Zhu Zhang, Wei Wei, Zi-Yun Chen, Xu Zhang, Tao Xiong, Zhen-Fei Wang, Luo-Yuan Xia, Jia-Fu Jiang, Wen-Jun Li, Dai-Yun Zhu, Na Jia, Wu-Chun Cao

**Affiliations:** 1 School of Public Health and Health Management, Gannan Medical University, Ganzhou, 341000 Jiangxi P. R. China; 2 State Key Laboratory of Pathogen and Biosecurity, Academy of Military Medical Sciences, Beijing, 100071 P.R. China; 3 Institute of EcoHealth, School of Public Health, Shandong University, 44 Wenhuaxi Street, Jinan, 250012 Shandong P.R. China; 4 School of Public Health, Fujian Medical University, Fuzhou, Fujian, 350122 China; 5 Guangdong Key Laboratory of Nanomedicine CAS-HK Joint Lab of Biomaterials, Shenzhen Engineering Laboratory of Nanomedicine and Nanoformulations Shenzhen Institute of Advanced Technology (SIAT) Chinese Academy of Sciences, Shenzhen, 518055 P. R. China; 6 Changchun Veterinary Research Institute, Chinese Academy of Agricultural Sciences, Changchun, China

**Keywords:** Biological techniques, Infection, Pathogens

## Abstract

Spotted fever group rickettsiae (SFGR) are obligate intracellular bacteria that cause spotted fever. The limitations of gene manipulation pose great challenges to studying the infection mechanisms of *Rickettsia*. By combining bioorthogonal metabolism and click chemistry, we developed a method to label *R. heilongjiangensis* via azide moieties and achieved rapid pathogen localization without complex procedures. Moreover, we constructed a C57BL/6 mice infection model by simulating tick bites and discovered that the stomach is the target organ of *R. heilongjiangensis* infection through in vivo imaging systems, which explained the occurrence of gastrointestinal symptoms following *R. heilongjiangensis* infection in some cases. This study offers a unique perspective for subsequent investigations into the pathogenic mechanisms of SFGR and identifies a potential target organ for *R. heilongjiangensis*.

## Abstract (web summary)

By combining bioorthogonal metabolism and click chemistry, we developed a method to label *Rickettsia heilongjiangensis* via azide moieties in vitro and in a C57BL/6 mice infection model by simulating tick bites.

## Introduction

Spotted fever group rickettsiae (SFGR) are obligate intracellular bacteria capable of causing a zoonotic disease known as spotted fever, primarily transmitted to hosts via tick bites^1^. The escalating prevalence and transmission of rickettsial diseases present a tremendous threat to public health. Among these diseases, *R. heilongjiangensis* is a newly discovered species of spotted fever group rickettsiae (SFGR), initially isolated from *Dermacetor silvarum* in 1983 in Heilongjiang province, China^2^. *R. heilongjiangensis* is a tick-borne pathogen causing spotted fever in the Far East, belonging to the *R. japonica* subgroup. Spotted fever caused by *R. heilongjiangensis* has been reported in Northeast China, Siberia, the Russian Far East, and Japan, resulting in numerous cases. Therefore, studying its pathogenic mechanism holds important public health importance.

Nevertheless, the constraints on gene manipulation and the application of shuttle plasmids pose a huge challenge to studying its infection mechanisms, given that *Rickettsia* is a strict intracellular parasite^3^. For example, plasmids have been identified predominantly in non-pathogenic *Rickettsia*, rather than in those causing severe diseases. Furthermore, partitioning and incompatibility of plasmids in rickettsiae may have adverse effects on the implementation of shuttle plasmid technology^4^. These factors present huge challenges to researching the pathogenesis of *Rickettsia*. Optical fluorescence labeling imaging is traditionally utilized as a vital approach to investigate virus-host interactions, employing bioluminescent reporter groups such as green fluorescent protein (GFP) and luciferase incorporated into the virus^5^, thereby detecting virus infection, replication, and transmission in vivo and in vitro. Nevertheless, integrating reporter groups into the virus is exceedingly challenging, and this fluorescent labeling technique may impact virus assembly and infectivity due to the chemical modifications of the virus capsid and envelope, potentially reducing its infectivity. Additionally, the incorporated fluorescent groups occupy surface recognition sites of the virus, thereby inhibiting virus adsorption on the virus receptor and entry into host cells.

Click chemistry is a rapid and efficient chemical synthesis method for molecules based on carbon-heteroatom connections, while bioorthogonal reactions are chemical reactions that adhere to the principles of click chemistry without interfering with organisms’ biochemical reactions^6–8^. The bioorthogonal click chemistry technique, which combines click chemistry and bioorthogonal reactions, is primarily employed for living imaging. Initially, this bioorthogonal click chemistry first introduces bioorthogonal chemical reporter molecules into target biomolecules through endogenous biosynthesis pathways (referred to as “metabolic labeling”). Subsequently, it covalently connects the exogenous probes with complementary groups to the chemical reporter molecules to visualize and track the target biomolecules. So far, the bioorthogonal metabolic labeling technique has been successfully applied to the labeling and imaging of biomolecules in bacteria^9^, viruses^10–15^, tissues^16^, and cells^17^. For instance, the exogenous fluorescent probe is covalently attached to azide moieties utilized by the virus during the replication process, enabling the observation of dynamic virus changes.

The reductive evolution of *Rickettsia* results in abundant adenine-thymine (AT) base pairs, highly conservative genomic sequences among species, and varying numbers of plasmids in *Rickettsia*^18,19^. A previous report suggests that reductive evolution deprives *Rickettsia* species to synthesize cell envelope glycoconjugates so that cell envelope glycans synthesis relies on host precursors to synthesize peptidoglycans (PGN) and lipopolysaccharide (LPS)^20^. Galactosamine is an amino derivative of the sugar galactose, which is a major component of glycoproteins, an essential part of living cells^21^. We speculated that *Rickettsia* might import it or its derivatives from the host cells. Thus, incorporating azide moieties into the host cell might enable *Rickettsia* to be functionalized with azide without direct chemical modification. Herein, we tried to label *Rickettsia* with an *R. heilongjiangensis* isolate available in our laboratory. N-azidoacetylgalactosamine-tetraacylated (AC4GalNAz) is azido derivative of N-acetyl galactosamine (GalNAc). AC4GalNAz could be used by the African green monkey kidney (Vero-81) when it was added to the culture medium. Subsequently, *R. heilongjiangensis* was inoculated into azide-labeled Vero-81 cells for growth and replication. Immunofluorescence assays demonstrated successful in vitro azide-labeling of purified *R. heilongjiangensis*, providing a unique perspective for in vivo tracking and subsequent target organ localization for *Rickettsia*.

## Results

### Assembly of *Rickettsia* isolate

The *Rickettsia* isolate utilized in this study was propagated in Vero-81 cells, Total DNA extraction was performed from Vero-81 cell cultures infected with the *Rickettsia*, and subsequent assessment of DNA yield and purity was conducted. Subsequently, the sequencing library was constructed following the whole-genome sequencing library construction protocol on the MGISEQ-2000 platform. Following whole-genome sequencing, we obtained more than 26 million 150-bp paired reads. Quality control was performed by AfterQC with 2.1% of reads and 9.1% of bases removed. After quality control, the high-quality clean reads were mapped to the genome of *Chlorocebus sabaeus*. The 96.55% of sequences mapped to the genome of *Chlorocebus sabaeus* were discarded. The remaining reads (3.45% sequences) were then de novo assembled into scaffolds using the SPAdes with default parameters. After binning and species annotation, a 1.33 Mb *Rickettsia* genome was obtained.

### Genomic characterization and phylogenetic tree construction

**Fig. 1:**
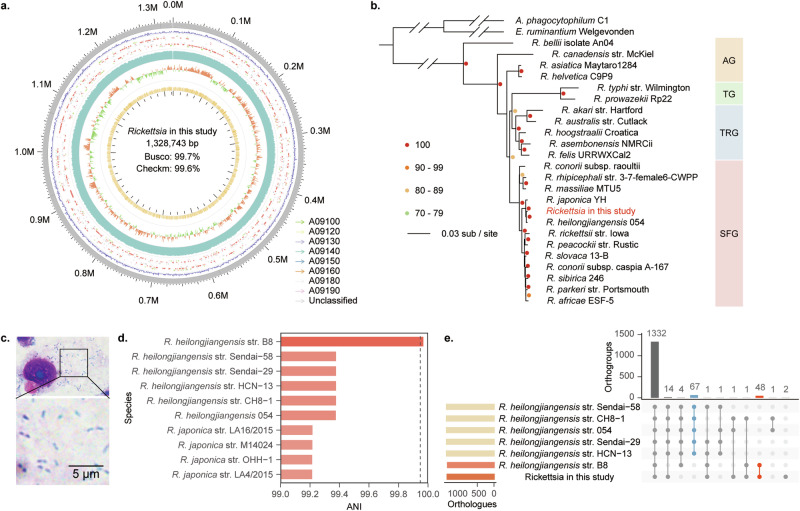
Identification of *Rickettsia* used in this study. **a** Bird’-eye view of the assembled genome of *Rickettsia* used in this study. The outer circle to the inner circle provides six types of information: contig length, gene density, gene annotation (arrow indicated direction color implies the KEGG of genes), DNA sequencing data coverage, GC skew value, and GC content. **b** The maximum likelihood phylogenomic tree of the *Rickettsia* used, along with 25 other publicly available Rickettsiales species. *Anaplasma phagocytophilum* and *Ehrlichia ruminantium* were two outgroup species used to root the tree. Four different groups of *Rickettsia*—spotted fever group (SFG), typhus group (TG), transitional group (TRG), and ancestral group (AG)—were indicated with different colors of backgrounds. **c** Giemsa staining of the *Rickettsia* used in this study in Vero-81 cells. **d** ANI between *R. heilongjiangensis* TIGMIC and other *R. heilongjiangensis* strains. **e** UpSetR plot showing orthogroups shared among different *R. heilongjiangensis* strains.

We used the whole purified culture for genome sequencing. The genome completeness of the *Rickettsia* isolate is evaluated at 99.7% by Busco and 99.6% by Checkm (Fig. 1a), with no duplicated BUSCO identified by Busco and 0.099% genome contamination evaluated by Checkm, indicating a very pure isolation. The guanine–cytosine (GC) contents of the *Rickettsia* in this study were 32.34%. The number of contigs in this assembled genome was 8, which indicates further improvement in assembly quality. The minimum and maximum contig lengths were 19,862 bp and 585,933 bp, respectively, with N50 and N75 of the genome being 176,561 bp and 138,911 bp, respectively. After genome annotation, 1,464 protein-coding genes, 33 tRNAs, and 3 rRNAs, were identified (Table 1). The phylogenetic tree was constructed as described in the Method section. The phylogenetic tree showed that *Rickettsia* isolate in this study was closely related to the reported *R. heilongjiangensis* (Fig. 1b). To verify the species of *Rickettsia* in this study, we extracted the sequences of six genes (*17* kDa, *gltA, groEL, ompA, ompB,* and *sca4*) from *Rickettsia* genome, and constructed the maximum likelihood tree. All the six maximum likelihood trees showed that *Rickettsia* isolate in this study was closely related to *R. heilongjiangensis* (Fig. S1). Therefore, we designated *Rickettsia* isolated in this study as *R. heilongjiangensis* TIGMIC (tick genome and microbiome consortium).

**Tab. 1:** Indicators of *Rickettsia* isolated in this study

Species	*R. heilongjiangensis*
Genome size (bp)	1,328,743
Busco	99.70%
Checkm	99.60%
GC content	32.34%
CDS	1464
tRNAs	33
rRNAs	3
No. of contigs	8
N50	176,561
N75	138,911
% coding	80.71%

### Identification of strains most closely related to *R. heilongjiangensis* TIGMIC

To identify the strains most closely related to *R. heilongjiangensis* TIGMIC, we calculated the average nucleotide identity (ANI) between *R. heilongjiangensis* TIGMIC with other Rickettsiae. An *R. heilongjiangensis* strain B8 was discovered to have highest ANI (Fig. 1d), and this strain was isolated from the serum of a patient who was bitten by a *Haemaphysalis longicornis* tick in Anhui Province, China^22^. After identifying the orthogroups among *R. heilongjiangensis*, *R. heilongjiangensis* TIGMIC uniquely shared 48 orthogroups with *R. heilongjiangensis* str. B8 (Fig. 1e), which is higher than the number it uniquely shared with other strains, suggesting its high similarity to *R. heilongjiangensis* str. B8 in proteins and its enormous potential to infect humans. This finding holds great epidemiological importance for the study of this bacterium.

### Azide moieties label Vero-81 cells but have no significant effect on cell viability

**Fig. 2:**
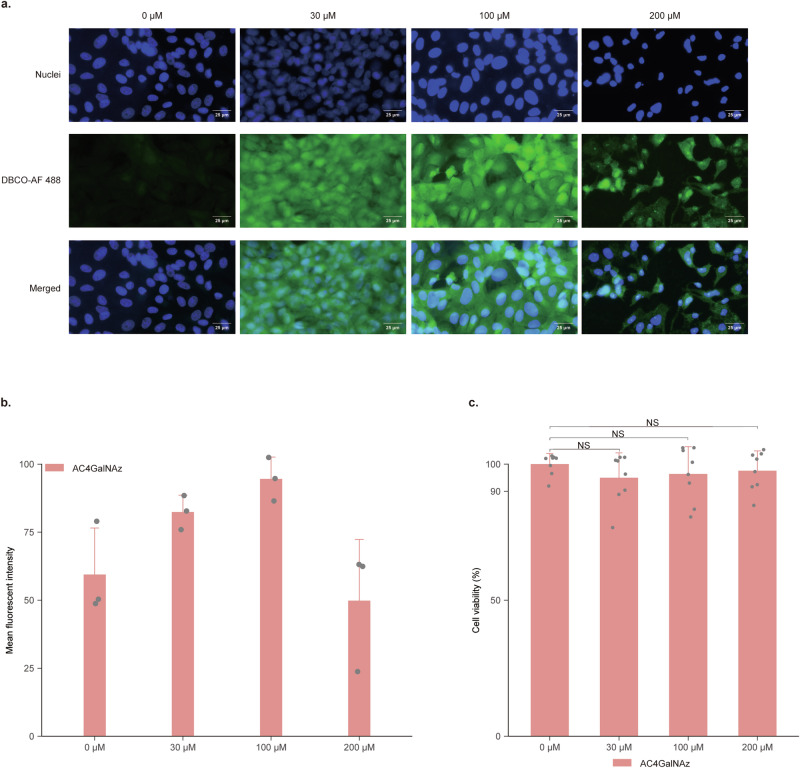
Labeling Vero-81 cells with azide moieties via bioorthogonal click chemistry. **a** Fluorescent images of Vero-81 cells labeled with azide moieties. Vero-81 cells were treated with 0, 30, 100, and 200 μM N-azidoacetylgalactosamine-tetraacylated (AC4GalNAz) for 24 h, respectively. After cell fixation, azide moieties and DBCO-AF 488 were covalently linked (green), and the nuclei were stained with Hoechst 33342 (blue). The fluorescence intensity of azide-labeled cells (green) was observed using a fluorescence microscope. Scale bar = 25 μm. **b** The mean fluorescence intensity of Vero-81 cells labeled with different concentrations of AC4GalNAz. Mean fluorescent intensity was calculated by ImageJ software according to the following formula. Mean fluorescent intensity = integrated optical density (IOD) ÷ total area of image. *n* = 3 biologically independent samples. Error bars represent geometric means ± SD. **c** Viability of Vero-81 cells labeled with different concentrations of AC4GalNAz. Vero-81 cells were treated with 0, 30, 100, and 200 μM AC4GalNAz for 24 h in 96-well plates. The cell viability was assessed using the CCK-8 assay. *n* = 8 biologically independent samples. Error bars represent geometric means ± SD. Mann–Whitney *U* test was used for comparison among groups. Vero-81 Cell viability with different concentrations of AC4GalNAz compared with without AC4GalNAz. *P* (30 μM) = 0.2258, *P* (100 μM) = 0.7984, *P* (200 μM) = 0.8785. NS, *P* > 0.05, no significance.

AC4GalNAz was added to the medium at various concentrations (0, 30, 100, and 200 μM) to enable their utilization by Vero-81 cells, thereby labeling the cells with azide moieties. 0 μM was the control group without adding AC4GalNAz. At 48 h post-azido labeling, Vero-81 cells were fixed and stained with DBCO-Alexa Fluor 488 (DBCO-AF 488). Galactosamine could be distributed in the cellular membrane, cytoplasm, and nucleus^21^. The results showed the utilization of AC4GalNAz by Vero-81 cells, resulting in successful cell labeling (Fig. 2a). The highest average fluorescence intensity for azide-labeled cells was observed at a concentration of 100 μM (Fig. 2b). To examine the effects of AC4GalNAz on cell growth and division, we assessed the viability of cells incubated with AC4GalNAz for 48 h. The cell viability remained above 90% at all four different concentrations (0, 30, 100, and 200 μM) with no significant difference compared with the control group (Fig. 2c). In conclusion, the above results indicated that AC4GalNAz effectively labels Vero-81 cells without significantly affecting cell viability, with the optimal concentration for labeling being 100 μM.

### *R. heilongjiangensis* can be labeled by azide moieties

**Fig. 3:**
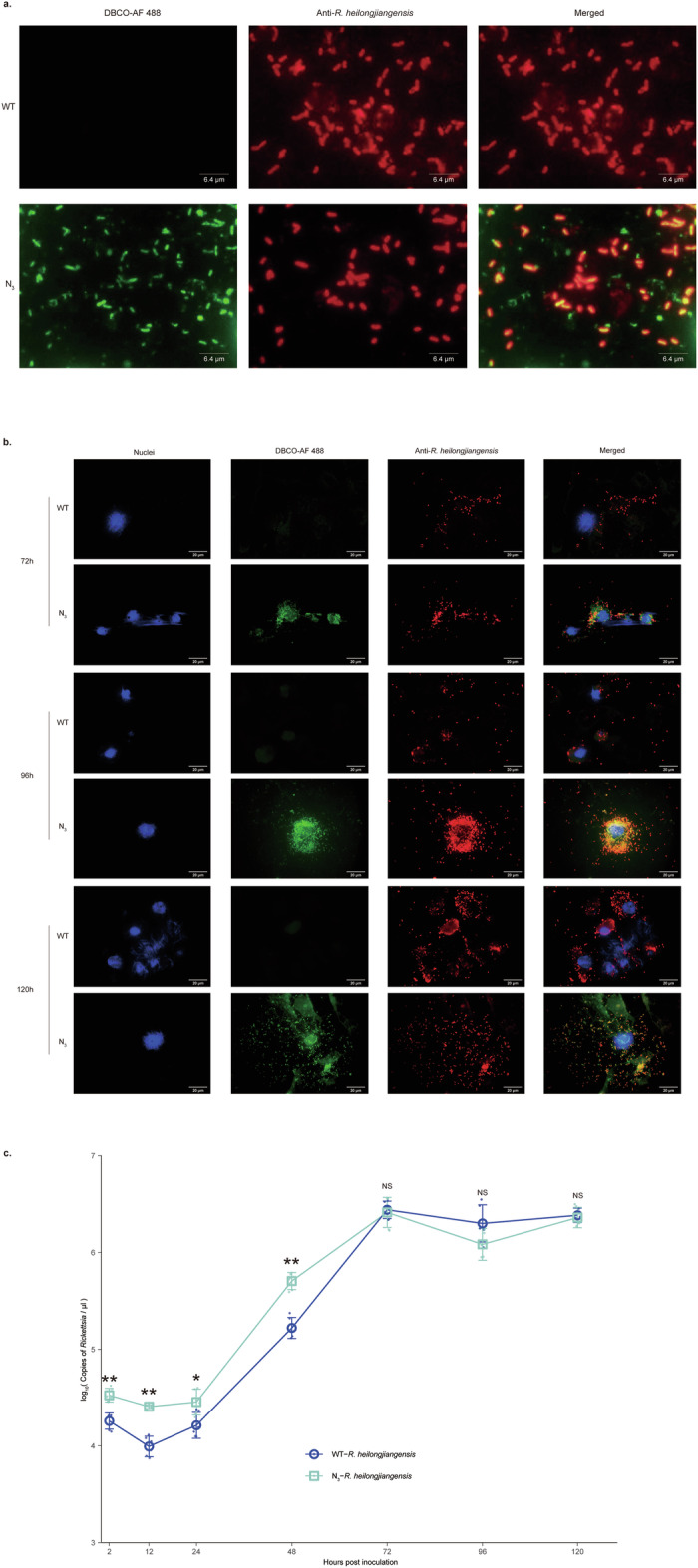
Infection and proliferation of N_3_-*R. heilongjiangensis.* **a** N_3_-*R. heilongjiangensis* was purified from azide-labeled Vero-81 cells, and it emitted green fluorescence signals after the reaction between DBCO-AF 488 and azide moieties through click chemistry. N_3_-*R. heilongjiangensis* was stained with mouse polyclonal antibodies against *R. heilongjiangensis* (red), then the fluorescence signals of DBCO-AF 488 (green) and *R. heilongjiangensis* (red) were observed by a fluorescence microscope. WT-*R. heilongjiangensis* served as the control. Scale bar = 6.4 μm. **b** Infection and proliferation of Vero-81 cells infected with N_3_-*R. heilongjiangensis* at 72, 96, and 120 h post inoculation (hpi). N_3_-*R. heilongjiangensis* was purified from azide-labeled Vero-81 cells, then infected Vero-81 cells on slides in 24-well plates. N_3_-*R. heilongjiangensis* emitted green fluorescence signals after the reaction between DBCO-AF 488 and azide moieties through click chemistry. Simultaneously, N_3_-*R. heilongjiangensis* was stained with mouse polyclonal antibodies against *R. heilongjiangensis* (red), and the nuclei were stained with Hoechst 33342 (blue) at 72, 96, 120 hpi, then DBCO-AF 488 (green) and *R. heilongjiangensis* (red) signals were observed with a fluorescence microscope. WT-*R. heilongjiangensis* served as the control. Scale bar = 20 μm. **c** Comparison of growth between WT-*R. heilongjiangensis* and N_3_-*R. heilongjiangensis* in Vero-81 cells. Growth curves of WT-*R. heilongjiangensis* and N_3_*-R. heilongjiangensis* in Vero-81 cells were analyzed using quantitative PCR. *n* = 3 biologically independent samples. Error bars represent geometric means ± 95%CI. The blue dots and lines indicate the copy number changes of WT-*R. heilongjiangensis*. The green dots and lines indicate the copy number changes of N_3_-*R. heilongjiangensis*. Mann–Whitney *U* test was used for comparison among groups. N_3_-*R. heilongjiangensis* compared with WT-*R. heilongjiangensis* at the same hpi. *P* (2 h) = 0.002, *P* (12 h) = 0.002, *P* (24 h) = 0.026, *P* (48 h) = 0.002, *P* (72 h) = 1, *P* (96 h) = 0.053, *P* (120 h) = 0.454. **P < 0.01; *P < 0.05; NS, *P* > 0.05, no significance.

As rickettsiae are obligate intracellular bacteria, they rely on metabolic substances provided by host cells^20^. In this study, *R. heilongjiangensis* was inoculated into azide-labeled Vero-81 cells. The purified *R. heilongjiangensis* strain was stained both with DBCO-AF 488 and a specific polyclonal anti-*R. heilongjiangensis* antibody. The results showed that green fluorescence signals emitted after click-chemical reaction with DBCO-AF 488 were co-localized with signals of *R. heilongjiangensis* when compared with wild type *R. heilongjiangensis* (WT-*R. heilongjiangensis*) (Fig. 3a). This indicates that purified *R. heilongjiangensis* could be labeled by azide moieties (N_3_-*R. heilongjiangensis*).

### Dynamic tracking of N_3_-*R. heilongjiangensis* in vitro

Spotted fever group rickettsiae (SFGR) replicate in the cytoplasm via binary fission, leading to serious damage to the ultrastructure of host cells by 48 h post-infection. SFGR also enter adjacent cells directly through actin-mediated polymerization, thereby altering the dynamics of the cell membrane and causing the fragmentation of damaged host cells^23^.

As a control, WT-*R. heilongjiangensis*, and the purified N_3_-*R. heilongjiangensis* strain was inoculated onto normal Vero-81 cells respectively. Subsequently, the cells were fixed and stained using click chemistry at various time points (72, 96, and 120 h post-infection (hpi)). The results indicated that after 72 hpi, some cells had ruptured, releasing large amounts of replicated *R. heilongjiangensis* to the outside of the cells, when some *R. heilongjiangensis* remained surrounding the nucleus in the cytoplasm. The DBCO-488 fluorescence signal of N_3_-*R. heilongjiangensis* was observed to be almost co-localized with the antibody signal of *R. heilongjiangensis* at 72, 96, and 120 hpi. (Fig. 3b).

To investigate the effects of azide-labeling on the growth and replication of *R. heilongjiangensis*, we inoculated N_3_-*R. heilongjiangensis* or untreated WT-*R. heilongjiangensis* into Vero-81 cells. The rickettsial load was then assessed at various time points (2, 12, 24, 48, 72, 96, and 120 hpi) using real-time quantitative PCR. *R. heilongjiangensis* exhibited slow replication and proliferation from 2 to 24 h, with proliferation peaking from 24 to 72 h. After 72 h, the proliferation rate began to decline, possibly due to the saturation of the number of *R. heilongjiangensis*, deterioration of infection, and decreased availability of nutrients in the medium. After 72 h, there is no significant difference between the growth of azide-labeled and wild-type *R. heilongjiangensis* (Fig. 3c).

### N_3_-*R. heilongjiangensis* can be tracked dynamically, and its target organs can be localized rapidly in a mouse infection model

**Fig. 4:**
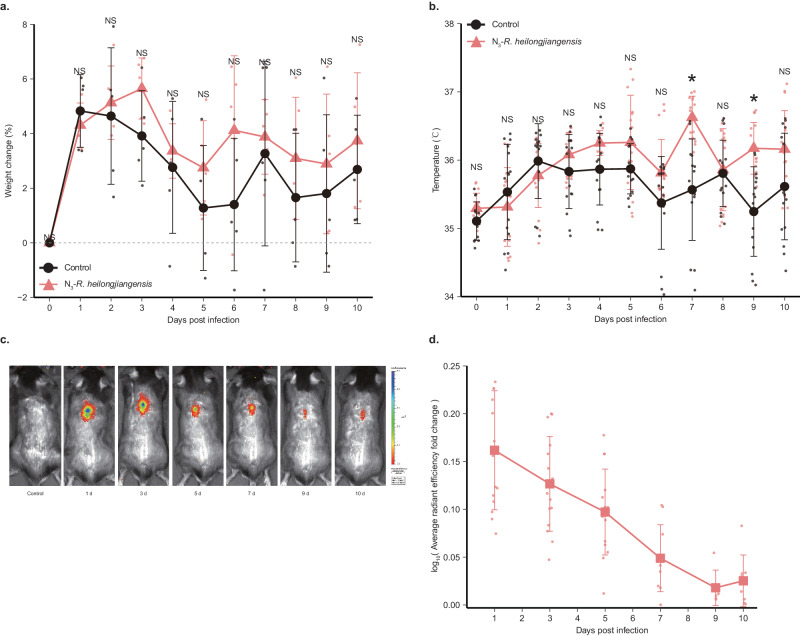
Construction of the infected mouse model. **a** Percentage of body weight changes of infected N_3_-*R. heilongjiangensis*-mice (1 × 10^7^ copies/μL) and control-mice through intradermal injection (i.d.). *n* = 5 biologically independent mice per group. Error bars represent geometric means ± SD. The black dots and lines indicate the weight changes of control-mice. The red dots and lines indicate the weight changes of N_3_-*R. heilongjiangensis*-mice. Mann–Whitney *U* test was used for comparison among groups. *P* (1 dpi) = 0.690, *P* (2 dpi) = 0.841, *P* (3 dpi) = 0.151, *P* (4 dpi) = 0.905, *P* (5 dpi) = 0.310, *P* (6 dpi) = 0.151, *P* (7 dpi) = 1, *P* (8 dpi) = 0.209, *P* (9 dpi) = 0.548, *P* (10 dpi) = 0.548. NS, *P* > 0.05, no significance. **b** Body temperature changes of infected N_3_-*R. heilongjiangensis*-mice (1 × 10^7^ copies/μL) and control-mice through intradermal injection (i.d.). *n* = 5 biologically independent mice per group. Error bars represent geometric means ± SD. The black dots and lines indicate the temperature changes of the control mice. The red dots and lines indicate the temperature changes of N_3_-*R. heilongjiangensis*-mice. Mann–Whitney *U* test was used for comparison among groups. *P* (1 dpi) = 0.548, *P* (2 dpi) = 0.207, *P* (3 dpi) = 0.310, *P* (4 dpi) = 0.413, *P* (5 dpi) = 0.421, *P* (6 dpi) = 1, *P* (7 dpi) = 0.036, *P* (8 dpi) = 0.917, *P* (9 dpi) = 0.047, *P* (10 dpi) = 0.548. NS, *P* > 0.05, no significance. **c** In vivo images of the dorsal side of C57BL/6 mice infected with N_3_-*R. heilongjiangensis* (1 × 10^7^ copies/μL) conjugated with DBCO Cy7 from day 1 to day 10 after i.d. The color bar is the reference for the radiance scale ([p/sec/cm^2^/sr]/[μW/cm^2^]). **d** Average radiant efficiency (average fluorescence intensity) of the dorsal side of C57BL/6 mice infected with N_3_-*R. heilongjiangensis* (1 × 10^7^ copies/μL) conjugated with DBCO-Cy7 through i.d. The average radiant efficiency was quantified using Living Image Software. Fold change of average radiant efficiency on the back of C57BL/6 mice. Fold change is the average radiant efficiency of N_3_-*R. heilongjiangensis* compared to WT-*R. heilongjiangensis* in infected mice. *n* = 15 biologically independent samples. Error bars represent geometric means ± SD.

**Fig. 5:**
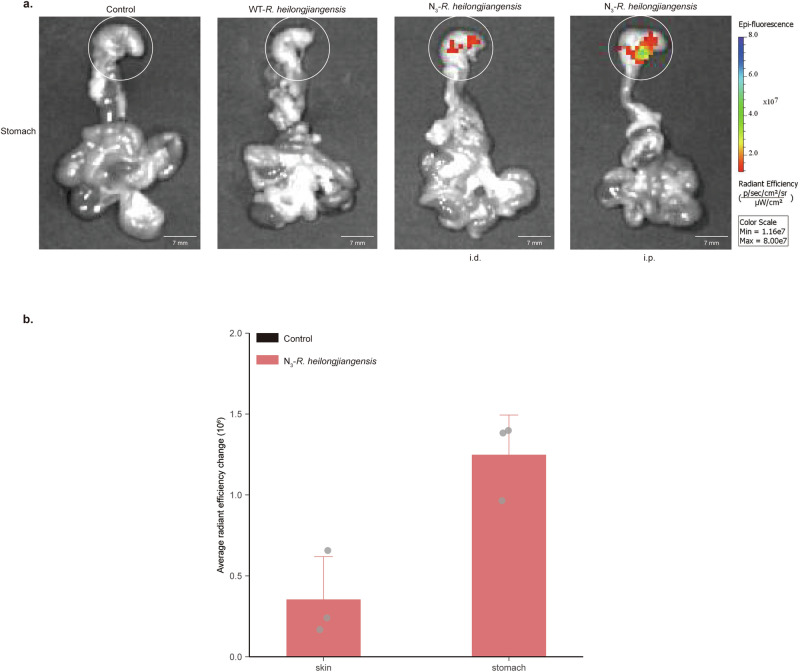
Localization of target organs by in vivo imaging. **a** Images of stomach of C57BL/6 mice infected with DBCO-Cy7-conjugated N_3_-*R. heilongjiangensis* on day 5 after intradermal (i.d.) inoculation and intraperitoneal (i.p.) inoculation. The stomachs of infected WT-*R. heilongjiangensis*-mice served as the control. *n* = 3 stomachs per group. The color bar is the reference for the radiance scale ([p/sec/cm^2^/sr]/[μW/cm^2^]). Scale bar = 7 mm. **b** Average radiant efficiency (fluorescence intensity) of skin and stomach on 5 days post infection (dpi) via intradermal (i.d.) inoculation. The average radiant efficiency was quantified using Living Image software. The black column indicates the average radiant efficiency of control-mice. The red column indicates the average radiant efficiency of N3-*R. heilongjiangensis*-mice. The change is the average radiant efficiency in the organs of N_3_-*R. heilongjiangensis* infected mice compared to WT-*R. heilongjiangensis* infected mice. *n* = 3 biologically independent samples. Error bars represent geometric means ± SD.

Additionally, we aimed to establish an in vivo imaging model of mice infected with N_3_-*R. heilongjiangensis*. DBCO-Cyanine 7 (DBCO-Cy7) was selected for its longer excitation wavelength, facilitating the visualization of N_3_-*R. heilongjiangensis* via in vivo imaging. DBCO-AF 488 was chosen for its stable wavelength allowing observation under fluorescence microscopy. N_3_-*R. heilongjiangensis* can proliferate rapidly after entering the mice, making it challenging for DBCO-Cy7/DBCO-AF 488 to accurately identify and bind to the azide moieties of N_3_-*R. heilongjiangensis* in vivo. Consequently, we opted for the pre-labeling method to deal with N_3_-*R. heilongjiangensis*. In vitro, the purified strain of N_3_-*R. heilongjiangensis* was pre-combined with DBCO-Cy7/DBCO-AF 488 and then used to infect C57BL/6 mice mimicking the transmission of *R. heilongjiangensis* by tick bites. We used C57BL/6 mice for intradermal (i.d.) injection way. The body weight and temperature of the mice was monitored daily. The results indicated there was no statistically significant difference in body weight between the experiment and control groups (Fig. 4a). The average body temperature of mice in the experiment group exceeded that of the control group from days 3 to 10. Notably, significant differences between the groups’ body temperatures were observed on days 7 and 9 (Fig. 4b). In vivo imaging of mice on days 1, 3, 5, 7, 9, and 10 revealed a gradual decrease in fluorescence intensity in the skin area on their backs over time (Fig. 4c, d). On the 5th day, mice were dissected and sampled, and their organs and tissues were imaged in vivo. Compared to control and WT-*R. heilongjiangensis* groups, fluorescence signals were detected in the stomach, indicating the presence of N_3_-*R. heilongjiangensis* in the mice’s organs and its replication on the 5th day, while no signals were observed in other organs (Fig. S2). N_3_-*R. heilongjiangensis* was reinjected into the groin, away from the stomach, via intraperitoneal (i.p.) injection to infect C57BL/6 mice. This resulted in N_3_-*R. heilongjiangensis* infecting stomach tissue via both injection methods on the 5th day (Fig. 5a). Additionally, the fluorescence intensity of the stomach was higher than that of skin via intradermal (i.d.) injection (Fig. 5b).

**Fig. 6:**
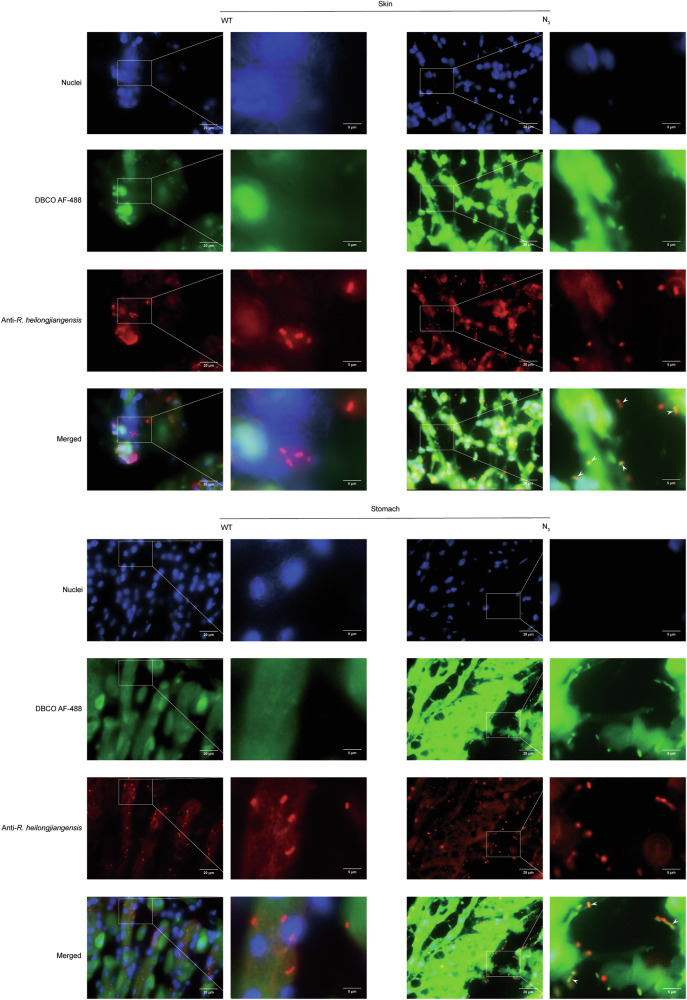
Distribution of N_3_-*R. heilongjiangensis* in infected tissues, including skin and stomach. The signals of cell nuclei stained with Hoechst 33342 (blue), DBCO-AF 488 (green), and immunofluorescence (red) were observed using a fluorescence microscope. The skin and stomach of infected WT-*R. heilongjiangensis*-mice served as the control. Solid white arrows indicated DBCO-AF 488 were co-localized with *R. heilongjiangensis* antibody signals. Scale bar of the original picture = 20 μm, scale bar of original magnification = 5 μm.

**Fig. 7:**
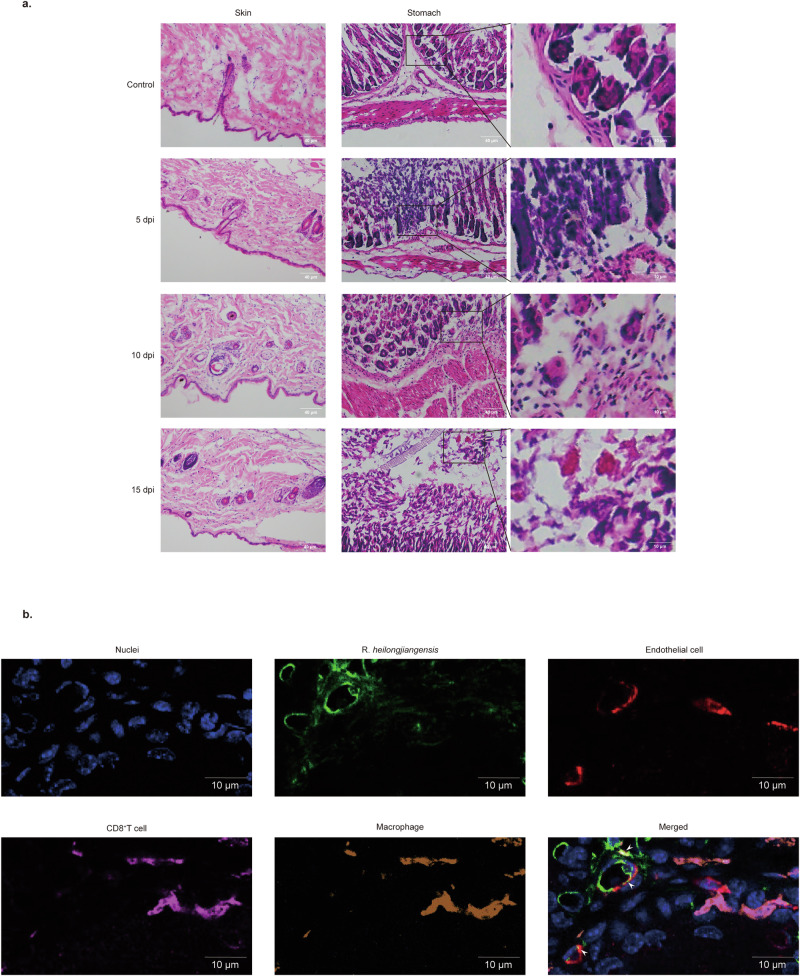
Inflammation in target organs. **a** Pathological changes of skin and stomach tissues of C57BL/6 mice infected with N_3_-*R. heilongjiangensis* through i.d. inoculation at 5, 10, and 15 dpi, as observed by hematoxylin–eosin (H&E) staining. Scale bar of the original picture = 40 μm, scale bar of original magnification = 10 μm. **b** Immunofluorescence images of gastric sections. The signals of cell nuclei stained with Hoechst 33342 (blue), *R. heilongjiangensis* (green), endothelial cell (red), CD8^+^T cells (pink) and macrophages (yellow). Solid white arrows indicated *R. heilongjiangensis* were co-localized with endothelial cells, CD8^+^T cells, and macrophages. Scale bar = 10 μm.

Skin and stomach samples were collected from infected mice, immediately fixed in 4% paraformaldehyde, and then sectioned into frozen sections. The results showed that green fluorescence signals emitted after click-chemical reaction with DBCO-AF 488 were co-localized with signals of N_3_-*R. heilongjiangensis* in frozen sections of skin and stomach, indicating that N_3_-*R. heilongjiangensis* can be traced in vivo. (Fig. 6). Following staining with hematoxylin and eosin (HE staining), the sections were observed under a fluorescence microscope. The skin did not show any distinct pathological changes. Infiltration of inflammatory cells of the gastric tissues developed at 5 days post-infection (dpi). Progressive damages in the stomach mucosal layer were visualized, with necrosis of the mucosa and multifocal hemorrhage at 15 dpi. No obvious histopathological change was observed in the stomach mucosa of control mice (Fig. 7a). The multiple immunofluorescence staining indicated that *R. heilongjiangensis* might be co-localized with the endothelial cells and macrophages (Fig. 7b). In conclusion, we successfully tracked N_3_-*R. heilongjiangensis* in vivo and localized its target organs in mice by combining azido groups with DBCO-Cy7/DBCO-AF 488 probe. This method offers a convenient and rapid approach for exploring the pathogenic mechanism of *R. heilongjiangensis* in future studies.

## Discussion

During reductive evolution, *Rickettsia* loses the genes associated with metabolic pathways, resulting in a small degraded genome (1.1–1.5 Mb), and the reduction of its genome correlates with increased pathogenicity^3,23–25^ and adaptability^26–29^. For example, the glycolysis pathway of *Rickettsia* is incomplete due to the lack of the genes required for the biosynthesis of endotoxins and peptidoglycans^30^, and these sugar components are not synthesized de novo but acquired from host cells. To compensate for the loss of genes, *Rickettsia* has developed a parasitic mechanism. Through this mechanism, numerous transport systems acquire the necessary metabolites from host cells to ensure the survival and replication of *Rickettsia*. For instance, active transport systems acquire adenosine triphosphate, amino acids, and phosphorylated sugars from host cells^31^. Since a large amount of sugar is needed in cellular growth and replication, azido sugars added to the medium can be undistinguishably utilized by host cells without significantly affecting cellular growth and vitality. Since *Rickettsia* is completely dependent on host cells for survival, the raw materials required for its glycometabolism can only be acquired from host cells, rather than from the culture environment. Therefore, we speculated that azide moieties could be introduced into *Rickettsia* through the process of glycometabolism. However, further work can give a profile on the glycosylation of metabolic glycan labeling of *Rickettsia* by tandem mass spectrometry with electron transfer dissociation fragmentation. In addition, the usefulness of another important sugar derivative, such as N-acetyl-glucosamine (GlcNAc), with this labeling method should be testified in the future.

It has been reported that 13 patients from the Russian Far East were diagnosed with acute rickettsial disease caused by *R. heilongjiangensis*. Among them, two patients exhibited nausea, and all 13 presented anorexia symptoms in 2002, suggesting a potential association between *R. heilongjiangensis* infection and damage to gastrointestinal blood vessels^32^. In 2014, All 733 hospitalized patients with severe fever with thrombocytopenia syndrome (SFTS) at a sentinel hospital in eastern central China exhibited multiple gastrointestinal manifestations, including anorexia, nausea, vomiting, and diarrhea^33^. These gastrointestinal symptoms have been reported in several types of spotted fever diseases, particularly in Rocky Mountain spotted fever (RMSF)^34,35^. We observed that the weight and body temperature of C57BL/6 infected mice did not exhibit significant changes. As previously reported in the literature, owing to the resistance of C57BL/6 mice to certain strains, there were no significant clinical symptoms such as changes in weight or body temperature, and these mice could not fully simulate the clinical symptoms of humans^36,37^. Though there are other mouse infection models, C57BL/6 mice models have the broad characteristics to elucidate the pathogenesis and immune mechanisms associated with human infection^38–40^. In addition, specific knockout animals can be obtained in the context of C57BL/6, which will enable future pathogenesis studies^41^. In general, non-specific background signals in the immunohistochemical staining are inherently difficult to diminished^42^, even though we used frozen sections. The azide-labeling technique is superior to the indirect immunofluorescence assay (IFA) for live cell labeling.

*Rickettsia* is transmitted to the host mainly through tick bites. After invading the target cells, *Rickettsia* escapes from the immune surveillance of the host cells and enters the cytoplasm. Facilitated by action induction, *Rickettsia* penetrates the cytoplasm of neighboring cells to establish infection^43,44^. Pathogenic rickettsiae mainly infects vascular endothelial cells, and the interactions between these pathogenic rickettsiae and their target cells lead to the secretion of inflammatory chemokines and cytokines, thus activating endothelial cells. Excessive activation of these immune responses can lead to tissue damage, potentially playing an important role in the pathogenesis of rickettsial diseases^45^. In this study, we real-time tracked N_3_-*R. heilongjiangensis*, and found that the pathogen might replicate in endothelial cells and release to the surrounding tissue^45^. The progressive damage in gastric mucosa suggests that the stomach may be the target organ of *R. heilongjiangensis*, and further investigations are needed to clarify the assumption.

## Methods

### Initial *R. heilongjiangensis* isolates from ticks

*R. heilongjiangensis* strain utilized in this experiment was isolated from *Haemaphysalis longicornis* collected from Anhui Province in 2019. The collected tick specimens were categorized based on features such basis capituli, scutum, pedipalp, gonopore, stigma plate, and other anatomical parts. Morphological analysis under microscopy identified the species as *Haemaphysalis longicornis*. Ticks testing positive for *R. heilongjiangensis* were isolated and cultured in vitro using Vero-81 cells (ATCC, Cat. No. CCL-81). The mixed ticks were immersed in 0.1% bromogeramine (Lircon, China) and 75% ethanol (Lircon, China), then rinsed with sterile PBS (Gibco, USA). After homogenization in 1 mL of Dulbecco’s Modification Eagle Medium (DMEM) (Gibco, USA) containing 2% fetal bovine serum (FBS) (Gibco, USA), 100 μL of the tick homogenate was inoculated into each well containing Vero-81 cells and incubated at 32 °C for 2 h in 24-well plates (Corning, USA). Subsequently, 1 mL of DMEM supplemented with 2% FBS, Penicillin–Streptomycin Solution (containing 100 U/mL penicillin, and 200 μg/mL streptomycin) (Solarbio, China) was added to each well. Media were changed, and cells were passed as scheduled. The strains remained stable and were preserved at low temperatures under laboratory conditions.

### Mammalian cell lines

The mammalian cell line utilized in the experiment was Vero-81, a kidney epithelial cell line derived from a female African green monkey (ATCC, Cat. No. CCL-81). Vero-81 cells were cultured in Dulbecco’s Modification Eagle Medium (DMEM) (Gibco, USA) supplemented with 10% fetal bovine serum (FBS) (Gibco, USA) at 37 °C in a humidified atmosphere with 5%CO_2_ and subcultured every 2–3 days. Vero-81 cells were cultivated in DMEM supplemented with 2% FBS when employed for *Rickettsia* species infection.

### Genome assembly and quality assessment

DNA extraction from the semipurified bacteria was performed using a High Pure PCR Template Preparation kit (Roche, Germany). Sequencing libraries were generated using the paired-end (PE) 150 bp strategy on the MGISEQ-2000 platform. Raw reads were filtered using the AfterQC v0.9.6 with default parameters. High-quality reads were aligned to the *Chlorocebus sabaeus* (GenBank assembly accession no. GCA_000409795.2) genome using the bowtie2 v2.4.1 with default parameters. The paired reads were discarded if one read matched the *Chlorocebus sabaeus* genome by using samtools v1.9 with parameters -f 12. Genome assembly was accomplished by SPAdes v3.15.2. Binning and genome reconstruction were accomplished by MetaBAT2 v2:2.15. Assembly quality was assessed through CheckM v1.1.3 in linage_wf mode and busco v4.1.2 with parameters -l rickettsiales_odb10.

### Genome annotation and construction of phylogenetic tree

Prokka v1.14.6 is used to annotate the gene with default parameters. Gene families were identified using orthofinder v2.5.4 with default parameters among 24 *Rickettsia* species and two outgroup species. Single-copy gene families (*n* = 347) were utilized for subsequent phylogenetic analysis. The protein sequences of these single-copy genes were aligned using MUSCLE v3.8.1551. Gblocks v0.91b was utilized to select conserved blocks from aligned sequences. Finally, iqtree v2.2.0 was used to generate a phylogenetic tree and calculate branch support.

### Genomic statistics summary and orthogroups identification

GC content and GC skew values were calculated using GCcalc v1.0.0. Next-generation sequencing reads were mapped to the final genome using bowtie2, and bedtools v2.30.0 was used to calculate the sequencing depth. KEGG annotation was applied using KofamScan v1.3.0. Average Nucleotide Identity with other *Rickettsia* species was calculated using fastANI v1.32. Orthogroup identification was fulfilled using orthofinder v2.5.4 with default parameters.

### Culture and purification of *Rickettsia heilongjiangensis* TIGMIC

To increase its quantity, frozen strains of *R. heilongjiangensis* were taken out of the liquid nitrogen and thawed in a 32 °C thermostatic water bath. A volume of 100 μL of *R. heilongjiangensis* suspension was inoculated into Vero-81 cells that had been cultured 24 h in advance per T75 flask (Thermo Fisher, USA), and then incubated in 5 mL DMEM medium supplemented with 2% FBS in a 32 °C incubator for 2 h. Subsequently, 15 mL medium was added per T75 flask, and *R. heilongjiangensis* was cultured in a 32 °C incubator with 5% CO_2_. The cells were observed daily. 4 days after inoculation, a *Rickettsia* purification experiment was conducted, obtaining the purified strains of *R. heilongjiangensis*. The *R. heilongjiangensis* load was detected using quantitative real-time PCR with specific primers (Sca1 Forward Primer for qPCR: GTTTGTGGATGCGTGGTA, Sca1 Reverse Primer for qPCR: AACCCGATAGTAGCAC).

The purified strains of WT-*R. heilongjiangensis* or N_3_-*R. heilongjiangensis* were obtained through a purification experiment. Infected Vero-81 cells were scraped off using a cell scraper (Biologix, China). The supernatant was transferred to a 50 mL centrifuge tube (Thermo, USA) using pipettes, and the tube was then placed on ice. The 27–30 gauge needle (Minank, China) was attached to the syringe, and the infected cell suspension was passed through it eight times to release the *Rickettsia*, the suspension was centrifuged at 1000xg for 10 min to precipitate the cell lysates. The supernatant was transferred to a 10 mL centrifuge tube (Thermo, USA) and centrifuged at 17,000xg for 10 min at 4 °C. The supernatant was discarded, and 1.8 mL of sucrose-potassium-glutamate (SPG) solution was used to resuspend the precipitate. The SPG solution contained 218 mM sucrose, 3.76 mM potassium phosphate monobasic, 7.1 mM potassium phosphate dibasic, and 4.9 mM potassium glutamate dissolved in distilled water, filtered through a 0.2 μm filters, and stored at 4 °C. The purified *Rickettsia* in SPG solution is suitable for in vitro experiments or preservation in liquid nitrogen. If used for in vivo infection, gradient purification is necessary. Pellets of *Rickettsia* were resuspended in 200 μL SPG, gently added onto 500 μL 25% N-Methyl-d-glucamine diatrizoate (Sigma, Germany), and precipitated by centrifugation at 17,000xg for 10 min at 4 °C to remove more cell debris, The appropriate amount of SPG was used to resuspend *Rickettsia* pellets according to the needs of the experiment^46^.

### Obtaining of N_3_-*R. heilongjiangensis*

The Vero-81 cells were passaged in advance to make them adhere to the wall surface. After 24 h, the medium was replaced with 100 μM AC4GalNAz (Click Chemistry Tools, USA) in DMEM medium supplemented with 10% FBS and the cells were incubated at 37 °C for 48 h. Then, the medium with AC4GalNAz was discarded, and the cells were washed with PBS for 2 times. Later, *R. heilongjiangensis* (1 × 10^7^ copies/μL) in DMEM medium supplemented with 2% FBS was inoculated into Vero-81 cells and cultured at 32 °C for 4 days. Then, the purified strains of N_3_-*R. heilongjiangensis* were obtained through the purification experiment described above. Unmodified Vero-81 cells were seeded into a 24-well plate (Corning, USA) and infected with either WT-*R. heilongjiangensis*) or N_3_-*R. heilongjiangensis* at a volume of 20 μL (1 × 10^7^ copies/μL) to assess *R. heilongjiangensis* infectivity in vitro.

In animal experiments, each experimental group of mice was injected with 50 μL (1 × 10^7^ copies/μL) of WT-*R. heilongjiangensis* or N_3_-*R. heilongjiangensis*, and the control group was injected with the same dose of PBS.

### Preparation of *R. heilongjiangensis* mouse polyclonal antibodies

For purpose of IFA experiments, it was necessary to prepare *R. heilongjiangensis* antisera as polyclonal antibodies. Five C3H/HeN mice (6–8 weeks old, male) (Beijing Vital River Laboratory Animal Technology Co., Ltd.) aged and 2 male New Zealand rabbits (Beijing Hua Fukang Biotechnology Co., Ltd.) weighing about 2 kg served as immunized animals, with *R. heilongjiangensis* administered via intraperitoneal and marginal ear vein injections, respectively. Each C3H/HeN mouse and New Zealand rabbit received 500 μL of *R. heilongjiangensis*, while the control group received an equivalent volume of PBS. The second immunization, utilizing the same method and dose, was performed 14 days after the initial administration. Seven days post-secondary immunization, blood was collected from the mice and rabbits via orbital and cardiac puncture, respectively, then stored at 4 °C overnight to precipitate serum. This serum was tested for antibody potency using the IFA assay and utilized as polyclonal antibodies in subsequent experiments.

### In vitro staining of Vero-81 cells and *R. heilongjiangensis*

Normal Vero-81 cells, N_3_-Vero-81 cells, WT-*R. heilongjiangensis*-infected Vero-81 cells and N_3_-*R. heilongjiangensis*-infected Vero-81 cells were tested by click reaction and immunofluorescence assay (IFA). The collected samples were fixed with 4% paraformaldehyde (MeilunBio, China) for 20 min at various time points (72, 96, and 120 hpi) and washed three times with phosphate-buffered saline (PBS) (Gibco, USA). The samples were incubated with DBCO-AF 488 (Click Chemistry Tools, USA, 1:500) at room temperature for 30 min in the dark, followed by three washes with PBS. The samples were immersed in 5% bovine serum albumin (BSA) (Solarbio, China) for 30 min to block non-specific binding sites. The *R. heilongjiangensis* mouse polyclonal antibodies (this paper, 1:200) were incubated at 37 °C for 1 h in the dark, followed by three washes with PBS. Alexa Fluor 594-labeled goat anti-mouse IgG secondary antibodies (Zsbio, China, 1:500) were allowed to incubate at room temperature for 1 h in the dark, followed by three washes with PBS. Coverslips (Citotest, China) were sealed by an anti-fade mounting medium with Hoechst 33342 (Invitrogen, USA)^47^. Fluorescent images were captured using a fluorescence microscope (Olympus, Japan)^14^.

### Cytotoxicity assay

Cell counting kit-8 (CCK-8) (Vazyme, China) was used to assess cell ability. After labeling the cells with different concentrations of AC4GalNAz in 96-well plates (Corning, USA) for 48 h, 10 μL of CCK-8 solution was added to each well (along the cell wall, gently shaken to avoid bubble formation), and cells were then incubated at 37 °C. After 2 h, cell viability was measured at 450 nm using a microplate reader (Thermo Scientific, USA)^48^.

### DNA extraction and quantitative real-time PCR

Cell samples were extracted using the MiniBEST Viral RNA/DNA Extraction Kit Ver.5.0 (TaKaRa, Japan). DNA was amplified using gene-specific primers and TB Green® Premix Ex Taq™ (Tli RNaseH Plus) (TaKaRa, Japan) in quantitative real-time PCR on a LightCycler® 480 II Instrument (Roche, Switzerland). The copy numbers of *Rickettsia* species were calculated using a standard curve method based on a plasmid containing the corresponding segment.

### Animal experiment ethics

C57BL/6 mice (6–8 weeks old, male), provided by Shanghai Model Organisms Center, Inc., were used for this study. All animal experiments were performed following the guidelines and regulations approved by the Institutional Animal Care and Use Committee (IACUC) of the Beijing Institute of Microbiology and Epidemiology. The animal ethics approval number is IACUC-DWZX-027-20. We have complied with all relevant ethical regulations for animal use. The mice were housed in a specific pathogen-free (SPF) environment. Isoflurane (Rwdls, China) was used as an inhalation anesthetic for mice prior to the experiment.

### Establishment of mouse infection model and sample collection

Initially, N_3_-*R. heilongjiangensis* was prepared by diluting it into cold sterile PBS buffer on ice, DBCO-Cy7/DBCO-AF 488 (Click Chemistry Tools, USA, 1:1000) was allowed to incubate at 32 °C for 40 min, and centrifugation at 17,000×*g* for 10 min at 4 °C. Subsequently, the DBCO-N_3_-*R. heilongjiangensis* was resuspended in precooled PBS to achieve the required volume (with a concentration of 1×10^7^copies/μL). Eight-week-old male C57BL/6 mice were divided into intradermal and intraperitoneal injection groups. Each type of injection was divided into three groups, with 6 mice in each group. The negative control groups were injected with 50 μL PBS, the positive control groups were injected with 50 μL of WT-*R. heilongjiangensis* (1 × 10^7^ copies/μL), and the experiment groups were injected with 50 μL of N_3_-*R. heilongjiangensis* (1 × 10^7^ copies/μL) each. Prior to injection, the back hair of the mice was shaved for ease of injection and in vivo imaging. Intradermal and intraperitoneal injections were administered using a 1 mL insulin syringe (BD Biosciences, USA). Body weight and temperature were monitored daily. Once no abnormalities were observed, the mice were returned to their cages for centralized feeding. Mice were dissected at 5 dpi, after which various organs were collected (Fig. S3). In vivo, imaging of dissected organs and immunostaining of tissue sections were performed according to the following descriptions.

### In vivo imaging

The images of live mice were recorded and analyzed on the 1, 3, 5, 7, and 9 dpi using an in vivo imaging system. Additionally, the dissected organs of the mice were imaged on the 5th days. The average radiant efficiency of the regions of interest (ROI) in mice was calculated in photons/s/cm^2^/steradian (p/s/cm^2^/sr) and analyzed using Living Image 3.2 software. We also conducted in vivo imaging of dissected organs using an in vivo imaging system (PerkinElmer, USA).

### Immunofluorescence and histology

The mice were dissected on the 5 dpi, the tissues were fixed in 4% paraformaldehyde (MeilunBio, China) at 4 °C. OCT-embedded tissues were sectioned at a thickness of 6 mm for immunofluorscence staining. Antigen retrievals were performed in citrate buffer (pH6) with a microwave (Midea, China) for 20 min at 95 °C followed by a 20 min cool down at room temperature. After three washes with PBST (PBS containing 0.5%Tween-20 (Solarbio, China)), the sections were incubated with 5% bovine serum albumin (BSA) (Solarbio, China) at 4 °C overnight to block non-specific sites. The *R. heilongjiangensis* rabbit polyclonal antibodies (this paper, 1:200) were allowed to incubate at 4 °C overnight, followed by three washes with PBST. The sections were then incubated with Alexa Fluor 594-labeled goat anti-rabbit IgG secondary antibodies (Zsbio, China, 1:500) at room temperature for 30 min. After three washes with PBST, coverslips (Citotest, China) were sealed by an anti-fade mounting medium with Hoechst 33342 (Invitrogen, USA).

### Hematoxylin–eosin staining

Frozen tissue sections were initially fixed in 4% paraformaldehyde (MeilunBio, China) for 1 min and subsequently washed once with distilled water. Subsequently, the sections were submerged in a staining vat containing hematoxylin (Beyotime, China), where they were stained for 5 min, and then washed with distilled water to remove the floating color. The sections were submerged in 1% acid alcohol fast differentiation solution (Beyotime, China) for several seconds, followed by a rinse with tap water to return to the blue, and a single wash with distilled water. Sections were then submerged in eosin staining solution (Beyotime, China) for 1 min, then washed with distilled water once. They were sequentially placed in different concentrations of alcohol from low to high and xylene for the purpose of transparency treatment. Finally, sections were sealed with neutral balsam (Solarbio, China) and observed under the microscope^45^.

### Multiple fluorescence immunohistochemistry (mlHC) assay

OCT-embedded tissues were sectioned at a thickness of 6 mm for immunohistochemistry staining. Multiplex fluorescence labeling was performed using TSA-dendron-fluorophores with NEON 5-color Allround Discovery Kit for FFPE (histovabio, USA). Briefly, frozen tissue sections were treated with 3% H_2_O_2_ (histovabio, USA) for 10 min to inactivate endogenous peroxidases, as followed by one wash with PBS. After incubation in blocking solution (2.5% normal goat serum (histovabio, USA)) for 20 min at room temperature, the slides were incubated with the *R. heilongjiangensis* rabbit polyclonal antibodies (this paper, 1:200) overnight at 4 °C. After three washes with PBS, the sections were incubated with ImmPRESS® HRP Goat Anti-Rabbit IgG Polymer secondary antibody (Vector Laboratories, USA) for 25 min at room temperature. DendronFluor® E-TSA (histovabio, USA) and Fluoreffer® Rapid Response Buffer (histovabio, USA, 1:200) were allowed to proceed at room temperature for 30–60 s for observation by microscopy. The sections were placed in ddH_2_O for 1 min, AbCracker® elution buffer (histovabio, USA) was allowed to cover the entire slide and incubate for 20 min at 37 °C. Then the sections were immersed in PBS for the next round of TSA staining, labeling different antigenic targets with different TSA fluorescence. The sections were incubated with nuclear dye (histovabio, USA, 1:400) for 10 min at room temperature. CrystalMount® Crystal Sealer (histovabio, USA) was allowed to cover the coverslips, then dried for more than 30 min at 37 °C.

Multiplex antibody panels applied in this study are CD31 (PECAM-1) (D8V9E) XP® Rabbit mAb (Cell Signaling Technology, USA, 1:300) for endotheliocytes, CD8α (D4W2Z) XP® Rabbit mAb for CD8^+^T cells (Cell Signaling Technology, USA, 1:300), F4/80 (D2S9R) XP® Rabbit mAb for macrophages (Cell Signaling Technology, USA, 1:300), ImmPRESS® HRP Goat Anti-Rabbit IgG Polymer secondary antibody (Vector Laboratories, USA). After all the antibodies were detected sequentially, the slices were imaged using a fluorescence microscope (Olympus, Japan).

### Statistics and reproducibility

All experiments were conducted at least three times. The *Rickettsia* load was quantified by the copy numbers of *Rickettsia* species (*n* = 3 biologically independent samples). The average radiant efficiency of the regions of interest (ROI) in mice was calculated in photons/s/cm^2^/steradian(p/sec/cm^2^/sr) and analyzed using Living Image 3.2 software (*n* = 5 biologically independent mice per group). The *Rickettsia* loads were quantified using qPCR. The *Rickettsia* loads were log-transferred, and presented as geometric means ± 95% CI. The average radiant efficiency was log-transferred, and presented as geometric means ± SD. Statistical differences between groups were determined using the Mann–Whitney *U* test and plotted in GraphPad Prism software 8.0 (GraphPad). A two-sided *p*-value of less than 0.05 was considered significant.

### Supplementary information

Supplementary information

## Data Availability

The data and code for analysis and visualization have been deposited to Zenodo. The whole genome of *R. heilongjiangensis* str. TIGMIC had been submitted to NCBI with BioProject PRJNA1050839.
