# Supplementary material for: Stomach as the target organ of Rickettsia heilongjiangensis infection in C57BL/6 mice identified by click chemistry
Source: Commun Biol. 2024 Jun 29;7:784. doi: 10.1038/s42003-024-06468-z (PMC11217389; doi:10.1038/s42003-024-06468-z)
Supplement: Supplementary file 1 — Supplementary information [file 42003_2024_6468_MOESM1_ESM.pdf]

Stomach as the target organ of *Rickettsia heilongjiangensis* infection in C57BL/6 mice identified by click chemistry

Fig. S1: Phylogenetic analysis of nucleotide sequence of genes in genome of *Rickettsia* used in this study and other *Rickettsia* species.

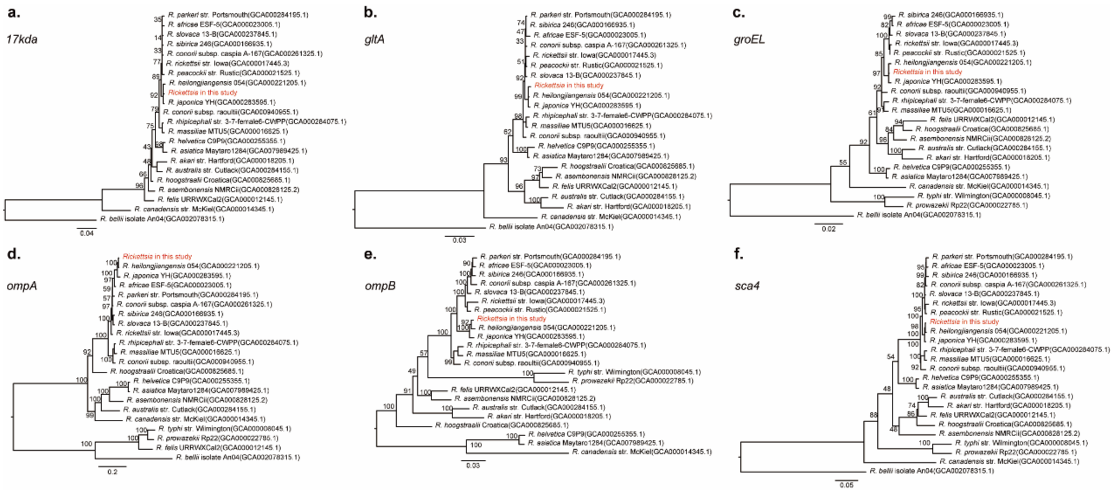

a 17*kDa* gene **b** *gltA* gene **c** *groEL* gene **d** *ompA* gene **e** *ompB* gene **f** *sca4* gene

Fig. S2: Images of C57BL/6 mice organs infected with DBCO-Cy7-conjugated N_3-*R. heilongjiangensis* on day 5 after intradermal (i.d.) inoculation.

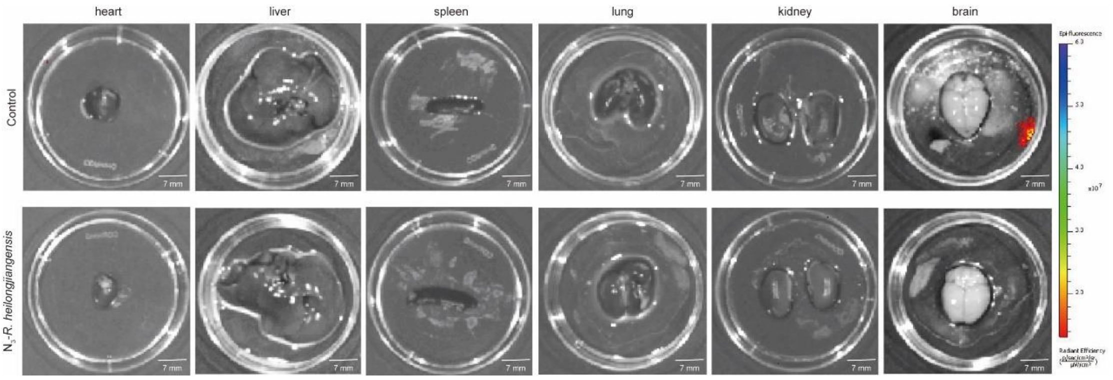

The color bar is the reference for radiance scale ([p/sec/cm^2 /sr]/[μW/cm^2]). Scale bar =7 mm.

Fig. S3: Experiment Scheme of Stomach as the target organ of *Rickettsia heilongjiangensis* infection identified by click chemistry. The figure was drawn by Figdraw (ID:UUUPAf979a).

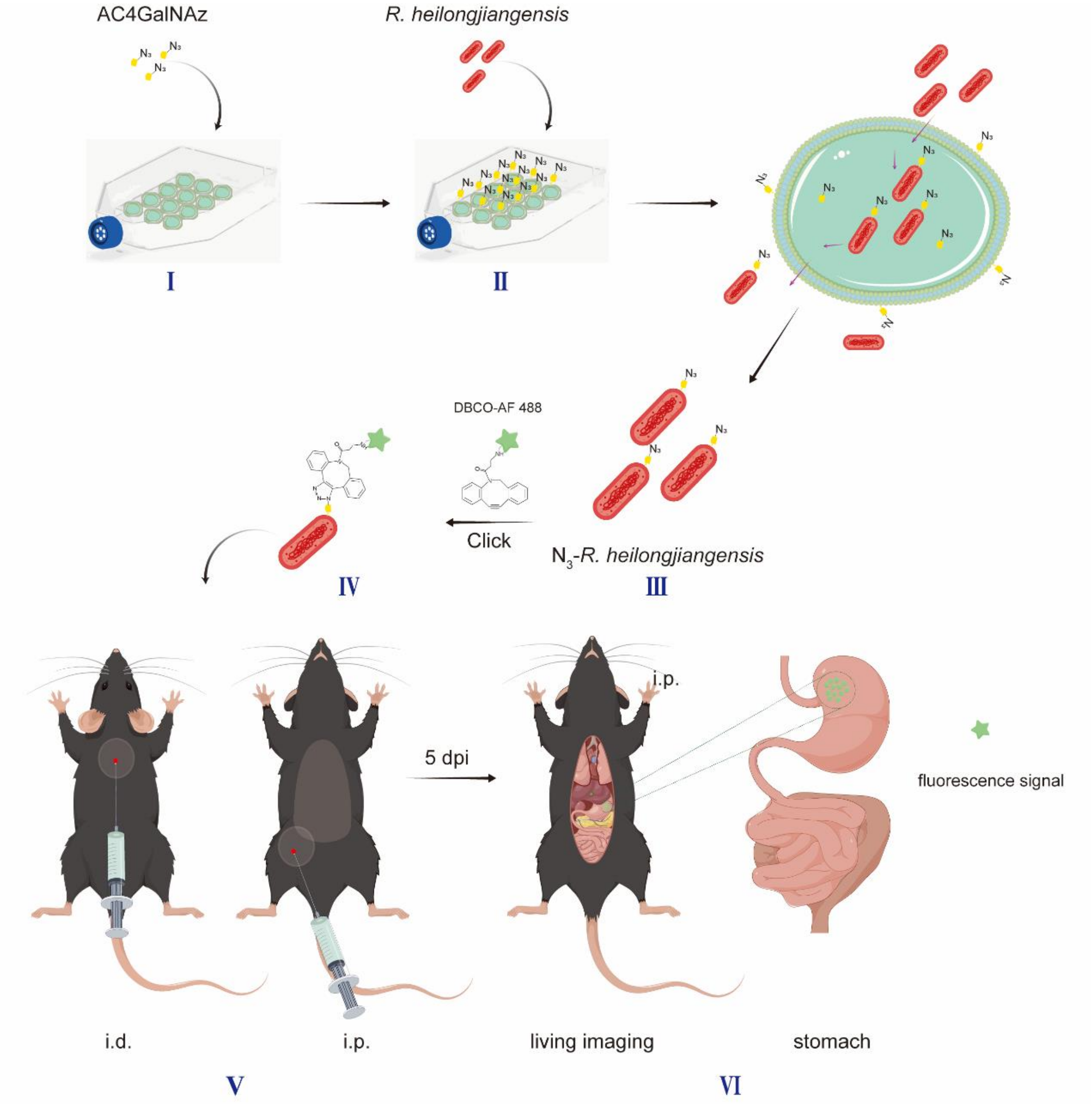

The labeling and tracking procedure as following: I) Labeling of host cells with Ac4GalNAz via bioorthogonal metabolism; II) Labeling of *R. heilongjiangensis* with Ac4GalNAz in Vero-81 cells; III) Purification of N_3-*R. heilongjiangensis* from infected Vero-81 cells; IV) Bioorthogonal click reaction with DBCO probes; V) Inoculation of DBCO-conjugated N_3-*R. heilongjiangensis* into mice; VI) *In vivo* imaging and observation of target organ stomach of *R. heilongjiangensis* infection.
